# Changes in the Color and Brightness of White Spots Associated with Orthodontic Treatment 6 Months after the Application of Infiltrative Resins: Systematic Review and Meta-Analysis

**DOI:** 10.3390/ijerph19159277

**Published:** 2022-07-29

**Authors:** Hugo Baptista-Sánchez, Laura Antonio-Zancajo, Alberto Albaladejo-Martínez, Pedro Colino Gallardo, Daniele Garcovich, Mario Alvarado-Lorenzo, Alfonso Alvarado-Lorenzo

**Affiliations:** 1 Department of Oral Surgery, Orthodontics Postgraduate, Universidad de Salamanca, 37007 Salamanca, Spain; baptistasanchez@gmail.com (H.B.-S.); albertoalbaladejo@usal.es (A.A.-M.); kuki@usal.es (A.A.-L.); 2 Department of Dentistry, Universidad Católica de Murcia, 30107 Murcia, Spain; pgo.pcolino@odontologiaucam.com (P.C.G.); malvarado@odontologiaucam.es (M.A.-L.); 3 Department of Dentistry, Universidad Europea de Valencia, 46010 Valencia, Spain; daniele.garcovich@universidadeuropea.es

**Keywords:** white spot lesion, orthodontic treatment, resin infiltration, lightness, colour

## Abstract

One of the risks that we find after orthodontic treatment is the secondary appearance of white spot lesions (WLS) after the removal of fixed multi-bracket appliances. Today, there are several treatment methods, resin infiltration being the most used in the most serious cases. The objective of this study is to carry out a systematic review and meta-analysis to determine the efficacy and stability in the variables of color and gloss, six months after resin infiltration. A comprehensive search was performed in the following databases: PubMed, Embase, Google Scholar, Scopus, Medline, and Web of Science. Articles published in the last 10 years were selected, including in vivo studies with a six-month follow-up. PRISMA guidelines were followed to carry out this systematic review. All studies where the application of resin was performed on carious lesions were discarded. Once the inclusion and exclusion criteria were applied, a final sample of four articles was obtained, on which the review and meta-analysis were carried out. Once examined, all authors considered that there was an immediate improvement in both variables. However, statistically significant differences were obtained in the color change outcome, but not in the brightness outcome in the subgroup analysis after six months of icon resin infiltration.

## 1. Introduction

Fixed multibracket orthodontics is the most widely used system in orthodontic treatments, whether by conventional, low friction or lingual brackets. Despite its multiple advantages in solving cases of dental malocclusions, it can also be associated with adverse side effects such as the appearance of lesions in the enamel, facilitated by the accumulation of plaque and oral bacteria in the bracket, the adhesive or the surface of the enamel itself [1,2,3].

These precarious lesions, in most cases, are whitish and opaque in appearance and are caused by demineralization of the enamel, called “white spot lesions” (WSL) [4] According to the different authors, the prevalence of WSL in orthodontic patients is between 11 and 97% [1,5,6,7] and may arise within one month of plaque accumulation, although the caries lesion needs at least 6 months to develop [5,8].

Most authors consider that they are more common in men than in women and may be associated with poorer hygiene in this group [7,9,10]. For Buschang et al., WSLs were more common in the maxilla than in the mandible [11]; however, other authors such as Khalaf et al. have found opposite results [12]. According to the dental location, we found that it is more frequent in upper and lower premolars, in upper anterior teeth and in lower molars [13,14], especially in the gingival areas or closer to the gingiva [12]. The percentage of patients with white spot lesions was lower in aligner wearers and higher in lingual orthodontics than in conventional brackets [11,15].

In the literature we found many preventive methods (before and during orthodontic treatment), although most authors consider that carrying out a prior assessment of caries risk before the start of orthodontic treatment would be the most advisable [16,17,18]. For this risk assessment, different systems have been used, such as plaque index measurements, photographs to determine pre-existing white spot lesions, saliva samples (Streptococcus mutans and Lactobacillus), cariogenic diet questionnaire and X-rays to determine the prevalence of caries [18,19], the latter method being the most recommended [18].

The intra treatment methods have included varnishes or toothpastes with chlorhexidine or fluorine [3,20,21], adhesives of brackets with or without bioactive fluorinated ceramic [22] use of nanoplatinum particles in sealants around the brackets, mouthwashes, pastes and dental adhesive [23,24,25], organoselenium sealants [26], etc. None of these methods seems to have a superior efficacy over the others in preventing the appearance of white spots during orthodontic treatment [5,25].

Once the white spot was established, different treatment methods were found in the literature. Some authors consider that if it is a mild form of WSL a natural remineralization could be attempted that would occur during the first year after the removal of the apparatus [27] or remineralization with fluoride varnishes or CPP-ACP paste (Casein PhosphoPeptide Amorphous Calcium Phosphate) [28,29,30,31,32]. For more advanced cases there are other methods, such as microabrasion or resin infiltration, and in severe cases composite resin restorations [33,34].

The resin infiltration technique for the treatment of white spots has been studied both in vitro and in vivo. It has a masking effect due to the high refractive index of the resin (1.44) unlike water (1.36) or air (1) substituted, and even lower than that of healthy enamel (1.63). After etching between 6% and 15% of the enamel surface with hydrochloric acid, the surface layer of the WLS is modified and the resin infiltrates, thereby modifying the degree of light scattering in the porous body of the WLS. injury [35,36]. Some authors consider that increasing the number of etchings in the enamel could improve the results [36]. The body of the lesion would also be waterproofed, helping to stop the diffusion of acids by creating a barrier within the lesion and not on its surface [37,38,39].

In this study we tried to analyze the efficacy and stability of the use of the infiltration resin, regarding the changes in the color and brightness of the post-orthodontic WLS at six months of treatment.

## 2. Materials and Methods

### 2.1. Search Strategy and Data Sources

In this study, a systematic review and meta-analysis were carried out following the guidelines established by the PRISMA guide [40,41]. To do this, the following specific questions were raised: How effective is resin infiltration for the treatment of WLS? How effective is stability at 6 months? How does this treatment affect the brightness and color of teeth with WSL?

The search was carried out independently by two authors (H.B.-S. and L.A.-Z.) and verified by a third author (A.A) between December 2021 and May 2022 in the PubMed, Google Scholar, Scopus, Embase, Medline and Web of Science databases using the search terms “White spot lesion; orthodontic treatment; resin infiltration; lightness; colour”.

### 2.2. Eligibility. Inclusion and Exclusion Criteria

The inclusion criteria for the selection of the articles were the following: (1) clinical trials that verified the effectiveness of resin infiltration (color and brightness) and had a minimum follow-up of 6 months, (2) patients with permanent dentition, (3) patients with conventional fixed orthodontic treatment who had it removed, (4) patients with post-orthodontic WLS who received a treatment for it by infiltration of resin (Icon^®^-DMG America, Englewood, NJ, USA), (5) articles published in the last 10 years, (6) articles published in English in journals with an impact factor and (7) “in vivo” articles performed on humans.

Those articles corresponding to reviews, case and control studies and monographic works were discarded. All articles referring to WSL treatment but using methods other than resin infiltration (Icon^®^) were discarded. All “in vitro” articles and those made in animals were also discarded. Additionally considered as exclusion criteria were those teeth that presented caries (cavitated or not), white spots derived from fluorosis or tetracyclines.

### 2.3. Study Selections and Data Extraction

For the selection of the articles used in this study, electronic and manual searches were carried out by two independent reviewers (H.B.-S. and L.A.-Z.) to guarantee the reliability of the results. Eligible studies were chosen by reviewing the title and published abstracts following the inclusion and exclusion criteria. For those titles not selected, the reason or reasons for their exclusion were stated. If during the process there was a disagreement for the inclusion of any work in the study, the opinion of a third reviewer (A.A.-L.) was taken into account.

The data selected for the study were: (1) author and year of publication, (2) review, (3) type of study, (4) number of affected teeth, (5) initial evaluation of brightness and color and (6) evaluation at 6 months of brightness and color.

### 2.4. Risk of Bias Assessment

**Figure 1:**
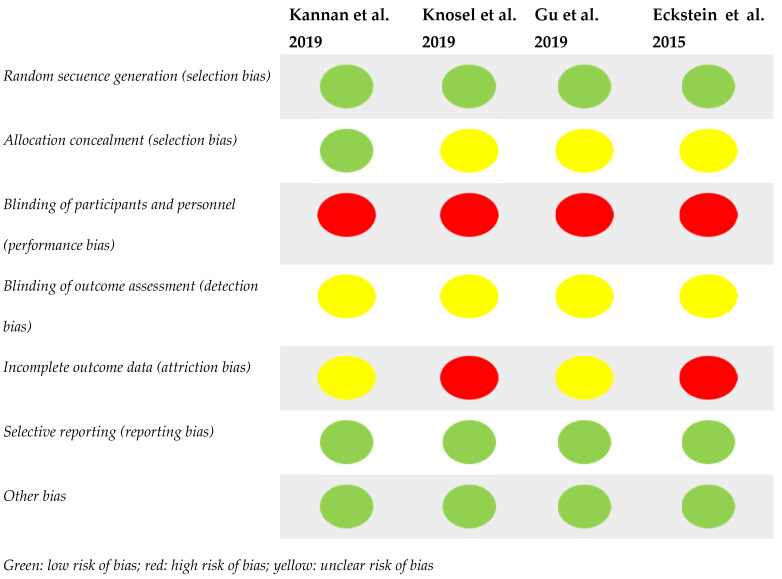
Risk of bias [36,43,44,45].

The risk of bias of this systematic review was determined by two independent reviewers (H.B.-S. and L.A.-Z.) and evaluated through specific scales, using the Cochrane risk-of-bias tool [42]. This tool evaluates selection bias, report bias, realization bias, detection bias, attribution bias, dropout bias and other biases of eligible items. Each of the biases was classified as high-risk bias, low-risk bias and unclear (Figure 1).

### 2.5. Data Analysis

There were two separate meta-analyses for each outcome (color and brightness). A fixed-effects model was adopted because of the evidence of homogeneity in the individual studies. A pooled effect size (mean difference) and a 95% confidence interval were assigned to each outcome. The Q statistic and I2 index were used to look at the effect size heterogeneity [46]. Results of the Q statistic (*p* < 0.01) indicated that the population was homogeneous nevertheless I2 index of heterogeneity of >90% indicated high heterogeneity. The IBM SPSS meta-analysis module (version 28.0) was used for all statistical study, setting the statistical significant difference threshold at 95%.

## 3. Results

### 3.1. Article Selections

**Figure 2:**
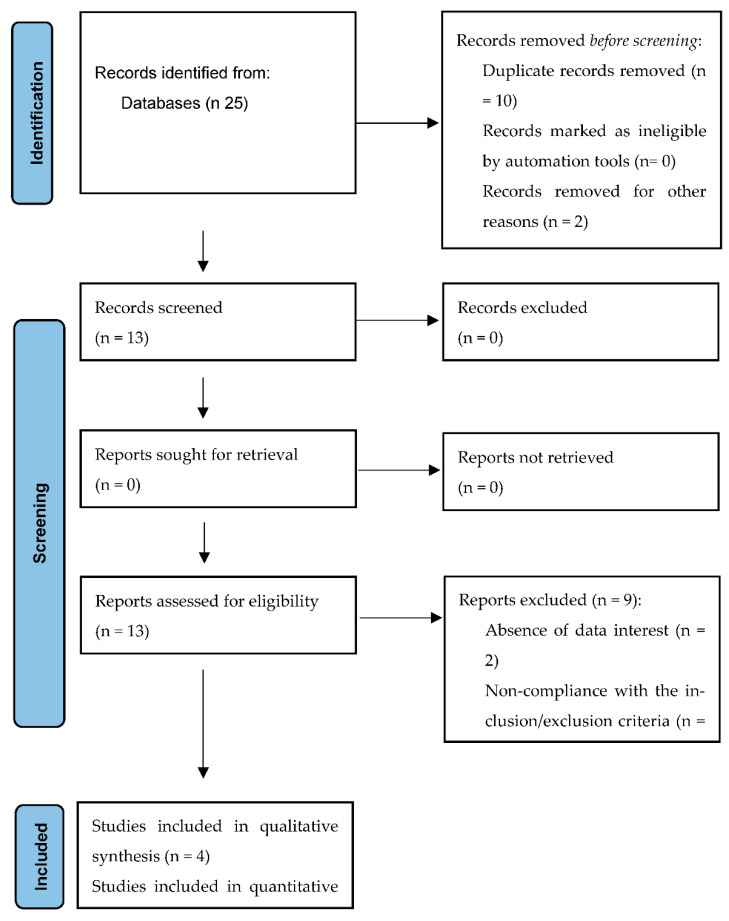
PRISMA flowchart.

After the initial review in the aforementioned databases, a total of 25 articles were obtained. A selection was made through the reading of titles and abstracts, obtaining a total of 13 eligible articles. The main causes for excluding the articles were the absence of data of interest, WLS not associated with orthodontic treatment, case–control studies, study of other treatments or analysis variables of the WLS and non-compliance with the inclusion/exclusion criteria. All the selected articles analyzed the infiltration of ICON resins in the treatment of white spots. Finally, four studies were selected for meta-analysis (Figure 2).

### 3.2. Study Characteristics

Table 1 and Table 2 shows the main characteristics of the studies included in this study. They were done between 2015 and 2019. After the selection of articles, four were included. Of these, four analyze the efficacy of the treatment taking into account the color variable [36,43,44,45] and three analyze the brightness variable [36,43,44].

The number of patients analyzed in each study was variable. We found that in the article by Kannan et al. [43] there were 12 patients analyzed, and in the articles by Gu et al. [45], Knosel et al. [44] and Eckstein et al. [36], the total number of initial subjects was 20 in each of them, reducing at the end of the study to 16 in the first case and 9 in the second two.

All of them analyze permanent teeth in general, only the Gu et al. study of 2019 focuses on anterior teeth [45].

### 3.3. Results of Meta-Analysis

#### 3.3.1. Color and Lightness Evaluation

**Figure 3:**
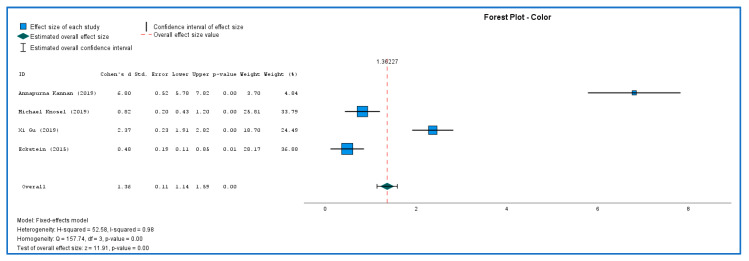
Color forest plot [36,43,44,45].

**Figure 4:**
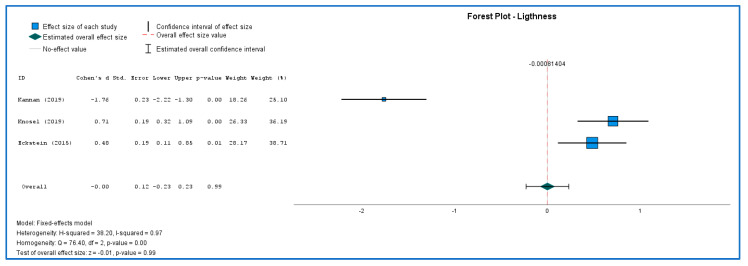
Lightness forest plot [36,43,44].

Figure 3 and Figure 4 shows the results of both studies. Because there were just a few studies, interpreting Q data required caution, and I2 index measures effect size heterogeneity more precisely. The forest plots were created to show the studies’ heterogeneity. Both color and lightness outcomes were heterogeneous. Each outcome was subjected to sub-group analyses to determine differences in effect sizes. The effect size of the color was statistically significant (MD = 1.36; 95% CI: 1.14 to 1.59; Z = 11.9; *p* < 0.01) but with a heavy outlier in Kannan article. Lightness (MD = −0.001; 95% CI: −0.23 to 0.23; Z = −0.007; *p* > 0.99) did not show statistical significance difference after 6 months of treatment.

#### 3.3.2. Risk of Bias

**Figure 5:**
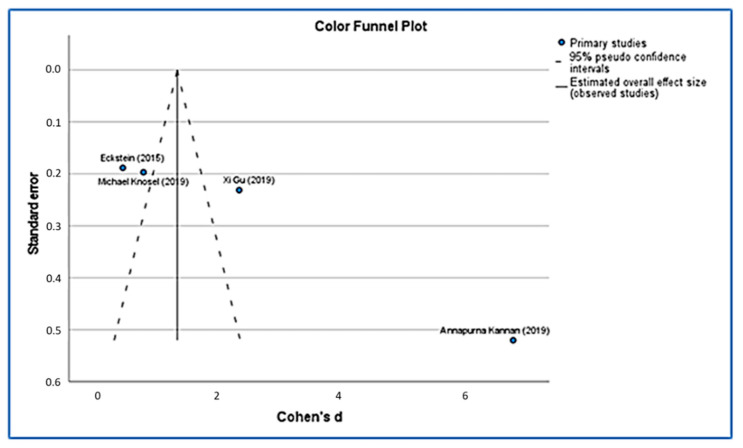
Color funnel plot [36,44,45].

**Figure 6:**
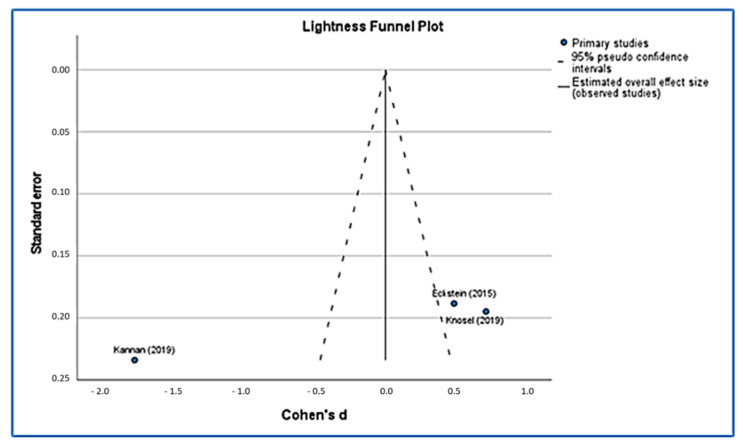
Lightness funnel plot [36,43,44].

Using funnel plots (Figure 5 and Figure 6), we were able to identify publication bias in the research, and our funnel plots show a few precisions. Their asymmetrical lines show high homogeneity and a high risk of bias. Poor methodological design, language bias and small sample sizes can also lead to this asymmetry.

## 4. Discussion

The main objective of this systematic review and meta-analysis was to analyze the efficacy at six months in the treatment of post-orthodontic white spots using low-viscosity infiltrative resins so that it could serve as a starting guide to choose the best treatment for our patients. For this, the variables change in color and brightness were evaluated six months after the application of the treatment.

After reviewing the literature, we found that one of the most commonly used treatments for WLS was queen infiltration and, in most cases, ICON (r) infiltrative resin [34,35,36,43,44,45,47]. We consider that analyzing this type of resins in our work could give us a broad view of the results in the WLS treatments of the same.

In the analyzed literature we found that the authors used different methods for the evaluation of the efficacy in the treatment of white lesions after the use of orthodontic appliances: changes in color, size, luminosity or fluorescence of the WLS. To evaluate the improvement in these characteristics, the different authors used independent or combined spectrophotometers [44,45,47], pre- and post-treatment photographs of the lesion [44,48,49] or devices such as diagnodent^®^ or vistacam^®^ to observe changes in enamel fluorescence [43,47].

The value of luminosity observed in white spot lesions is of great importance when evaluating the efficacy of possible treatments. There is a difference in the refraction value between healthy enamel and enamel with WLS: this value is lower in white spot lesions because light is absorbed and scattered differently in the micropores of the lesion. Therefore, an increase in luminosity and refractive index would indicate an improvement in white spot injury [50,51,52].

In the study by Kannan et al., 2019, where they compare the use of resin infiltration (ICON) with the use of a fluorinated varnish (Cilinpro XT^®^), they observe how there is a greater change in luminosity at first after the application of the same, probably related to the use of initial acid etching that would have allowed the varnish to penetrate deeper inside the lesion by removing its surface layer [43]. Other authors consider that the improvement effect with the use of fluoride pastes or varnishes works best with a formulation combined with phosphates and calcium, as in the case of Remin Pro^®^, since they are able to fill the superficial lesions of the enamel [29,47]. On the other hand, it seems that the color properties initially improve more with the infiltration of ICON^®^ resin thanks to the presence of Bis-GMA and the resin itself [36,43].

We found a study that analyzes and compares the changes in WLS after the application of infiltrated resin (ICON^®^) and microabrasion, obtaining a better result in both color and brightness with the first treatment [45]. It seems that the infiltration of low-viscosity resin in the lesion would improve its appearance and size and would also prevent the progression of caries, since it would prevent the entry of acid into it. On the contrary, microabrasion would irreversibly wear the surface of the lesion [45].

It seems that the surface hardness, dimensions, and depths together with the surface roughness of the WLS would influence the final aesthetic results in the treatment. Those with more favorable conditions would need fewer etching intervals and would improve the depth of infiltration, and therefore long-term aesthetics [36].

It has been proven that the changes in color and brightness are maintained over time (12–45 months of study) after infiltration with resins (ICON^®^), improving the aesthetics of teeth with postorthodontic white spot [36,44,45] without producing negative side effects in patients after infiltration, so it would be a good method of camouflage of these white spots [45].

None of the authors consulted assesses in their results whether these changes in color or brightness may be related to the inhibition of the progression of WLS to caries lesions, an aspect that we consider important. It would be interesting to analyze in possible studies both the aesthetic and clinical aspects regarding the evolution of the lesion to caries in the short and long term after infiltration with WLS resins without good patient hygiene [34,35,36,43,44,45,47].

To carry out this work, 25 studies were evaluated, and finally, 4 studies were included in the meta-analysis (4 that assessed changes in color and 3 that assessed changes in brightness) with a total of 458 teeth analyzed. In all the articles analyzed, the authors suggest that both the gloss and the color would improve after the infiltration of low-viscosity resins [36,43,44,45] and that these results would be stable over time [36,44]. After analysis by subgroups, a great heterogeneity was found in the variables. The results suggest that there are no statistically significant differences in the groups in the gloss variable that occurred in the WLS after six months of treatment with low-viscosity infiltration resins. However, differences were found at the color level, probably due to the atypical values found in the study by Kannan [43] compared to the other authors [36,45,47]. These results could be conditioned by the limitations at the time of carrying out this work; therefore, more studies should be carried out to analyze these variables and treatment in a more homogeneous way.

## 5. Limitations

The first limitation of the present study is related to the limited number of studies included. This is due, on the one hand, to the multitude of treatments for white spots with different formulations (varnishes or pastes with fluoride, sodium fluoride, CPP-ACP; infiltration with resins, microabrasion, etc.) and, on the other hand, to the lack of consensus in the way they are used. Furthermore, we found it difficult to analyze these since many authors use the number of patients as variable *N* (independently of the number of teeth with WLS present in each one) and others the number of teeth with WLS (independently of the number of patients used). We have used the number of teeth as a sample since not all the articles included in this work specified the number of white spots analyzed.

In addition, we found a variability in the comparative time analyzed in the different studies. Some authors analyze the changes in color and brightness in the first month, others between 0 and 3 months or even from 0 to 6 months with intervals in between. Therefore, it has been decided to analyze only the common times that would include the changes between T0 (start of treatment) and T1 (six month after start of treatment). We consider that it would be necessary for a better evaluation of the efficacy in changes in color and brightness of the WLS, on the one hand to expand the sample size of the work and, on the other hand, to expand the number of time moments analyzed in order to be able to evaluate if differences in brightness and color at other time points (for example immediate time, one month, three months) so that we can obtain a time line of changes.

## 6. Conclusions

After the comparative evaluation of the changes in brightness and color in white spots derived from orthodontic treatment after six months of the use of low-viscosity infiltration resins (ICON^®^), the authors agree on an improvement in both in brightness as in color. No statistically significant differences were found between the groups in the luminosity variable, but they did exist in the color variable. Due to the limitations in terms of the heterogeneity of results, methodology and low sample of studies found, more long-term studies are necessary and with a more homogeneous methodology.

## Figures and Tables

**Table 1:** Summary of the study characteristics of the records.

Author and Year	Journal	Study Type	Type of Treatment	Tooth Type	Sample (Teeth)	Assessment Criterion	Follow-Up	Results
Kannan (2019) [43]	Prog. Orthod	RCT	Icon^®^ resin infiltration Clinpro xt Varnish^®^	Incisor, canine, premolars, first molar	102	Color, Brightness	3, 6 months	The immediate results were better with resin infiltration, but at 3/6 months the situation was reversed.
Knosel (2019) [44]	Angle Orthod.	RCT	Icon^®^ resin infiltration Control Group	Permanent teeth.	111	Color, Brightness	6, 12, 24, 45 months	Improved long-term WLS with stable results.
Gu (2019) [45]	Angle Orthod.	RCT	Icon^®^ resin infiltration Microabrasion	Permanent anterior teeth.	128	Color	1 week, 6, 12 months	Similar results in both methods, slightly superior with resin infiltration.
Eckstein (2015) [36])	Angle Orthod.	RCT	Icon^®^ resin infiltration	Permanent teeth	117	Color, Brightness	1, 6, 12 months	Color and brightness improve after resin infiltration and results are stable in the long run.

RCT: randomized controlled trial.

**Table 2:** Summary of brightness and color values.

	Group	Teeth	Lightness				Colour					
			T0 (before Intervetion)		T1 (6 Month after Intervetion)		T0 (before Intervetion)		T1 (6 Month after Intervetion)		Change t0–t1	
			Mean	SD	Mean	SD	Mean	SD	Mean	SD	Mean	SD
Kannan (2019) [43]	Icon resin infiltration	102	74	0.8	81.88	6.27					9.66	1.42
Knosel (2019) [44]	Icon resin infilltration	111	73.9	4.84	70.96	3.36	9.12	5.63	5.5	2.76	3.61	
Gu (2019) [45]	Icon resin infiltration	128					6.57	2.48	2.20	0.82		
Eckstein (2015) [36]	Icon resin infiltration	117	72.55	3.45	70.88	3.49	8.15	3.74	6.33	3.81	1.81	

## Data Availability

Data are contained within the article.
